# Development of bispecific antibodies with enhanced neutralization activity against tested SARS-CoV-2 Omicron subvariants

**DOI:** 10.3389/fimmu.2026.1793368

**Published:** 2026-06-17

**Authors:** Ching-Hsuan Hsu, Ting-Yi Chang, Ya-Min Chang, Shin-Han Lin, Tzu-Ning Chen, Keng-Hao Hsu, Jhong-Jhe You

**Affiliations:** AP Biosciences Inc., Taipei, Taiwan

**Keywords:** bispecific antibody (bsAb), COVID-19, neutralizing antibody, Omicron strain, SARS-CoV-2

## Abstract

**Background:**

The COVID-19 pandemic, driven by the SARS-CoV-2 virus, has posed significant global health challenges, exacerbated by the emergence of the highly mutable Omicron variant. This study explores the potential of bispecific antibodies (bsAbs) in neutralizing this variant more effectively compared to traditional monoclonal antibodies (mAbs) and their combinations.

**Materials and methods:**

A comprehensive approach was utilized involving a fully human antibody phage-display library, Omni-Mab, to identify and enrich phage clones specific to SARS-CoV-2. Recombinant spike receptor-binding domain (RBD) proteins from the Omicron variant and other strains served as antigens during the biopanning process to ensure a broad screening. Post-enrichment, the binding affinity of the antibodies to the spike proteins of various strains was rigorously evaluated. Antibodies demonstrating strong binding efficacy were strategically combined and engineered into bsAbs. The neutralizing efficacy of these bsAbs was subsequently tested using various strains of the SARS-CoV-2 pseudo-viruses.

**Results:**

Selected parental mAbs showed kinetic profiles ranging from 5.71 x 10–^10^ to 2.53 x 10–^4^ M across the tested Omicron subvariants, and the lead bsAbs, particularly R3-1a-1/R4-1a-10 and R4-21/R4-1a-51, demonstrated enhanced binding activity and lower IC50 values than the controls and mAb cocktails.

**Conclusion:**

The research unveiled two highly promising bsAbs, namely, R3-1a-1/R4-1a-10 scFv and R4-21/R4-1a-51 scFv. These bsAbs showed strong binding and neutralizing activity across the tested Omicron subvariants and retained activity against selected non-Omicron pseudovirus strains evaluated in this study.

## Introduction

The COVID-19 pandemic has dramatically impacted global health, presenting numerous challenges across epidemiological, medical, and socioeconomic dimensions. The emergence of the Omicron variant of severe acute respiratory syndrome coronavirus 2 (SARS-CoV-2) further complicated the course of the pandemic because of its extensive antigenic divergence from earlier strains (1, 2). Characterized by a high mutation burden, enhanced immune evasion capabilities, and increased affinity for the angiotensin converting enzyme-2 (ACE-2) receptor, Omicron and its descendant sublineages have posed substantial challenges to both vaccine protection and antibody-based therapies (1, 3–7). These virological features contributed to rapid global spread and repeatedly reduced the effectiveness of pre-existing immune interventions, highlighting the need for antibody strategies that can better tolerate ongoing antigenic drift (1, 2, 8).

In response to these challenges, antibody-based therapeutics have remained an important component of the anti-SARS-CoV-2 treatment landscape. Combination therapies composed of two or more monoclonal antibodies (mAbs) can improve protection relative to single-antibody monotherapy by simultaneously engaging multiple viral epitopes and reducing the likelihood of immediate mutational escape (9–12). However, antibody cocktails still face notable limitations, including increased manufacturing complexity, higher production cost, and declining effectiveness as the virus accumulates escape-associated mutations (13).

Bispecific antibodies (bsAbs) have therefore emerged as an attractive alternative format for antiviral antibody engineering. By incorporating two binding specificities into a single molecule, bsAbs may enhance functional potency, broaden reactivity, and improve resistance to antigenic variation compared with monospecific antibodies in selected settings. Recent SARS-CoV-2 bsAb studies have employed diverse architectures, including IgG-like bispecific formats, IgG-scFv fusion designs, tetravalent bsAbs, and bispecific single-domain antibodies (12, 14–18). In some of these reports, the improved performance of bsAbs was supported by epitope-informed design, structural analysis, or direct evidence for simultaneous and synergistic engagement of two spike epitopes, whereas other studies extended bsAb breadth to more immune-evasive Omicron descendants and even related sarbecoviruses (13, 14). Collectively, these studies underscore the promise of bsAb engineering, while also showing that antiviral performance depends strongly on molecular format, parental antibody pairing, and the specific variant panel used for evaluation (18).

In this study, B.1.1.529, BA.2, BA.4, and BA.5 were selected as representative tested Omicron subvariants because BA.1/B.1.1.529 and BA.2 represented major early Omicron branches, whereas BA.4/BA.5 emerged later and showed greater antibody evasion than earlier Omicron sublineages (3, 5–7, 19, 20). In contrast to studies centered on epitope-defined or structure-guided bsAb optimization, the present study used phage-display screening as an accessible and cost-effective discovery approach to identify fully human parental antibodies, particularly in the absence of convalescent clinical samples or single-cell antibody isolation platforms. The main contribution of this study lies in the generation and functional evaluation of bsAb candidates derived from these selected parental antibodies, and in assessing whether this engineering strategy improves *in vitro* binding and neutralization performance across a defined panel of tested Omicron subvariants.

## Materials and methods

### Enrichment of SARS-CoV-2 specific phage clones

To enrich SARS-CoV-2-specific phage clones from a fully human antibody phage-display library called Omni-Mab (AP Biosciences), recombinant SARS-CoV-2 spike RBD His-tag proteins (Omicron strain wild type (WT), N484K/N501Y of WT, E484K of WT, L452R/E484Q of Delta strain, and L452R/T478K of Delta strain) (Acrobiosystems, Newark, Delaware, USA) were employed as antigens during the panning process. Different antigens were respectively coated on 96-well Nunc-Immuno Maxisorp plate at 4 °C overnight. The solution in each well was discarded and the well was washed three times with 0.4 ml of 0.1% Tween-20 in phosphate buffered saline (PBST). These antigens on plate were then blocked with 5% skim milk in PBS at room temperature for 1 hours. After washing with PBST, approximately 10^10^ plaque-forming unit (PFU) phages from Omni-Mab libraries in 0.1 mL PBS were added to each well and incubated for 1 hour at room temperature. The wells were washed with PBST for 3 times to remove unbounded phages. Phages binding to antigens specifically were eluted by triethylamine (TEA) and neutralized in 1 M Tris-HCl buffer pH 8.0. Half of the eluted phages were used to infect TG1 competent cell in the mid-log phase to amplify the phage and prepare for another round of screening or enrichment. The other half were used for propagation and polyclonal phage enzyme-linked immunosorbent assay (ELISA). After three or four rounds of biopanning, individual phage clones were isolated for the assessment of their binding activity using ELISA. Subsequently, phagemids from the selected phages displaying specific binding activity were isolated and sequenced to verify the integrity and diversity of the VH and VL fragments.

### ELISA assay

Collected phages from selected clones were added to the antigen-coated wells and incubated for 2 hours at 37 °C. After washing with 350 μl of PBST, 100 μl anti-M13 mAb conjugated to horseradish peroxidase (HRP) (Amersham-Pharmacia-Biotech, Vienna, Austria) was added at a final concentration of 1/8000 dilution. The microplate was incubated for 1 hour at 37 °C. Tetramethylbenzidine (TMB) substrate was added after washing and the reaction was stopped with 3N sulfuric acid after 15 min. Color intensity was measured at 450 nm by ELISA reader.

### Cell lines

ExpiCHO cells were purchased (Thermo Fisher Scientific, Schwerte, Germany). ExpiCHO cells were cultured and maintained in ExpiCHO™ Expression Medium (GIBCO, Thermo Fisher, Waltham, MA, USA) according to the manufacturer’s protocol. The optimal cell density ranged between 4×10^6^ and 6×10^6^ cells/mL in a shake flask. This cell line contributes to incubate on an orbital shaker platform at 37 °C under 8% CO2. For expanding the cells, the shake speed was set to 125 ± 5 rpm.

### Generation and purification of SARS-CoV-2 specific monoclonal antibodies

The DNA fragment of variable region in selected phage clones were amplified and cloned into an IgG expression vector (AP Biosciences) that carrying the IgG1 constant region. Plasmids encoding both heavy chains and light chains were transfected into ExpiCHO cells (Thermo Fisher Scientific, Schwerte, Germany) for mAb production in the form of IgG. After 6 days of culture, harvest cell culture fluid (HCCF) was affinity purified from culture supernatant by Protein A chromatography. Purified antibody was concentrated, followed by dialysis in PBS buffer. The antibody concentration was determined using a NanoDrop2000 spectrophotometer. The purified antibodies were analyzed by sodium dodecyl sulfate-polyacrylamide gel electrophoresis (SDS-PAGE) with Coomassie blue staining under non-reducing and reducing conditions to assess their electrophoretic profiles and apparent molecular weights.

### Construction of bispecific antibody

The bsAb against SARS-CoV-2 was constructed by using one of the selected clones in IgG1 and another selected clone in the form of single chain fragment variable (scFv) which was fused to the C-terminal of the constructed IgG1 (**Supplementary Figure 1**). A short flexible peptide linker of (GGGGS)_4_ was placed between the C-terminal of IgG1 and N-terminal of the scFv to ensure correct folding and minimize steric hindrance. Constructed bsAb was expressed using the Gibco ExpiCHO Expression System and purified from the cell culture supernatant of transfected cells via 1-step Protein G chromatography. **Supplementary Figure 1** shows the structural representation of the symmetric format of anti-SARS-CoV-2 bsAb (**Supplementary Figure 1**).

### SDS-PAGE analysis

Antibodies were analyzed by NuPAGE^®^ 4-12% gradient gels (ThermoFisher, Cat. NP0321BOX). The concentration of antibodies was determined using NanoDrop2000 spectrophotometer with A280 absorbance (Thermo Scientific, Rockford, USA). Antibodies were prepared for non-reducing and reducing condition as manufacture’s instruction. Prepared reducing samples with 25 mM DTT were heated at 70°C for 10 min, and immediately cooled on ice. After running and band separation, gels were stained with Coomassie blue staining reagent and destained with ddH_2_O. The images were photographed in visible light view.

### HPLC analysis

The aggregations and quality of concentrated anti-SARS-CoV-2 bsAb was measured by high performance liquid chromatography (HPLC). HPLC was performed using a Waters ACQUITY Arc system with Waters 2489 UV/Vis detector. Samples were load into BioResolve SEC mAb Column (Waters, Cat#186009441) with isocratic 25 mM sodium phosphate, 200 mM NaCl, pH 6.8 as mobile phase for protein separation. The flow rate was 0.4 mL/min and the sample injection amount was 40 μg. Peaks were detected by absorbance at 280 nm. All loaded samples were filtrated with 0.22 μm filter (Millipore, Cat#SLGV004SL) to remove any precipitated protein material. Data were analyzed by Empower 3 software.

### Antibody integrity evaluation

Antibody integrity evaluation was carried out by microfluidic capillary electrophoresis (μCE-SDS) with Protein Clear HR Assay on a LabChip GXII instrument (Perkin Elmer, Inc.). According to the manufacture’s protocol, 2.5 μL of protein sample at 1 mg/mL was mixed with 18 μL of sample buffer. The sample buffer was prepared by mixing 700 μL of Protein Clear HR sample buffer with either 24.5 μL of 1M DTT (for the reducing assay) or 24.5 μL of 0.25 M NEM (for the non-reducing assay). Prepared samples were incubated at 70°C for 10 min. After cooling to room temperature, 35 μL of water was added to each sample before loading onto the instrument. The samples were then analyzed using the Protein Clear HR Assay script. Meanwhile, the chip was prepared as instructed by the manufacturer and maintained at 30°C throughout the analysis. The excitation and detection wavelengths are 635 nm and 700 nm, respectively.

### Binding affinity assay

To perform a direct ligand binding assay of anti-SARS-CoV-2 antibody, recombinant SARS-CoV-2 spike proteins stated before were pre-coated on the Nunc-Immuno Maxisorp 96 well plates and incubated at 4°C overnight. Excess protein was removed by washing three times with 0.4 ml of PBST. The 0.4 ml blocking solution (5% non-fat milk powder in PBS) was added to all wells and incubated at room temperature for 1 hour. The blocking buffer was removed and washed three times with 0.4 ml PBST. Pre-coated wells were incubated with serial dilutions of purified anti-SARS-CoV-2 antibodies in 0.1 ml PBS for 1 hour at room temperature. The antibody solution was removed and the plates were washed three times with 0.4 ml PBST. HRP-conjugated goat anti-human IgG, F(ab’)_2_ specific antibody (Jackson Immunoresearch #109-036-097) was diluted 1:2000 with PBS and added at 0.1 ml per well. The plates were incubated for 1 hour at room temperature and washed three times with 0.4 ml PBST per well. HRP substrate TMB (Invitrogen) 0.1 ml were added into wells and incubated for 1 to 5 minutes at room temperature. 0.05ml 1N HCl was added to stop the reaction and the absorbances were read at 450 nm on a Bio-Tek Spectra. For the relative binding analyses, the ELISA binding signal of each tested antibody was normalized to that of Bebtelovimab, which was set to 1 for each Omicron subvariant separately.

### Binding kinetic analysis

The binding kinetics has been analyzed by FortéBio biolayer interferometry (BLI). Dip and Read Anti-Human IgG Fc Capture (AHC) biosensors (Sartorius) in a 96-well plate format (F-Bottom microplates, Greiner) with a baseline buffer consisting of kinetics buffer (DPBS with 0.05% Tween 20/0.2% BSA). Each mAb was loading at 5 μg/mL onto the AHC biosensor followed by associated with two-fold serial dilution of Omicron spike proteins and dissociated with kinetic buffer. When different concentration recombinant spike proteins interacted with mAbs in solution, we obtained the changes in the kinetic data via altering the phase shift of the light wave. The dissociation constant of anti-SARS-CoV-2 antibody binding to SARS-CoV-2 spike protein was calculated using Octet Data Analysis HT software (Sartorius).

### Neutralization assay

The neutralizing activities of constructed antibody against ACE2 receptor or pseudovirus was tested. For the ACE2 neutralization assay, purified antibodies were dialyzed in PBS and ACE2 were conjugated with biotin (abcam #ab201795). Nunc-Immuno Maxisorp 96 well plates were pre-coated with recombinant SARS-CoV-2 spike proteins, and incubated at 4°C overnight. Coated wells were blocked with blocking buffer (PBST containing 1% BSA) at room temperature for 1 hour. After washing three times with 0.35 mL PBST, serial 2-fold dilutions of anti-SARS-CoV-2 antibody (from 30 to 0 nM) and a fixed concentration (5.88 nM) of biotin-conjugated ACE2 in blocking buffer were added and incubated at room temperature for 1 hour. The antibody-ACE2 mixtures was removed and washed three times with 0.35 mL PBST. The HRP-Streptavidin solution was added and incubated at room temperature for 1 hour. After washing three times with 0.35mL of PBST, 0.1 mL TMB solution was added and incubate for 5 minutes until color development. The reaction was stopped by 0.05 mL 1N HCl, and the absorbance at 450nm was measured by SpectraMax iD3 microplate reader. The percentage of neutralization was calculated by the following formula: Neutralization (%) = (OD450nm of ACE2 alone - OD450nm of antibody lead+ACE2)/OD450nm of ACE2 alone) × 100%. Half maximal inhibitory concentration (IC50) was calculated by nonlinear regression using GraphPad Prism (GraphPad Software Inc., La Jolla, San Jose, CA, USA) software.

For the pseudovirus neutralization assay, a lentiviral-based system was employed, consisting of packaging plasmids, a transfer vector encoding a firefly luciferase reporter, and a SARS-CoV-2 Omicron (B.1.1.529) Spike protein envelope plasmid (Abnova, Catalog #KA6152). The assay was performed using these lentiviral pseudoviral particles. Serially diluted antibodies were pre-incubated with the pseudovirus at room temperature for 30 min before addition to 293T-hACE2 cells. After **48** h of incubation, cells were lysed and luciferase activity was measured using the Luciferase Assay System (Promega, #E1501) on a Biotek Synergy LX Multi-Mode Reader.

The percentage of neutralization was calculated as the reduction in luciferase signal (relative light units, RLU) compared to virus-only control wells. IC50 values were determined by variable-slope nonlinear regression using GraphPad Prism software. Assays were performed in biological duplicate. For cocktail comparisons, the two parental mAbs or the commercial comparator antibodies were mixed at a fixed 1:1 molar ratio, and the total antibody concentration of each cocktail was matched to that of the corresponding bsAb at each dilution point. All comparator and bsAb samples were evaluated in parallel under the same assay conditions.

## Results

### Isolation and characterization of SARS-CoV-2 spike RBD-specific antibody fragments from phage display library

Via the Omni-Mab phagemid library, the mAb phagemid clones showed significantly higher binding activity compared to controls, indicating effective enrichment of phage clones targeting the SARS-CoV-2 spike RBD protein variants (**Supplementary Figure 2**).

**Figure 1:**
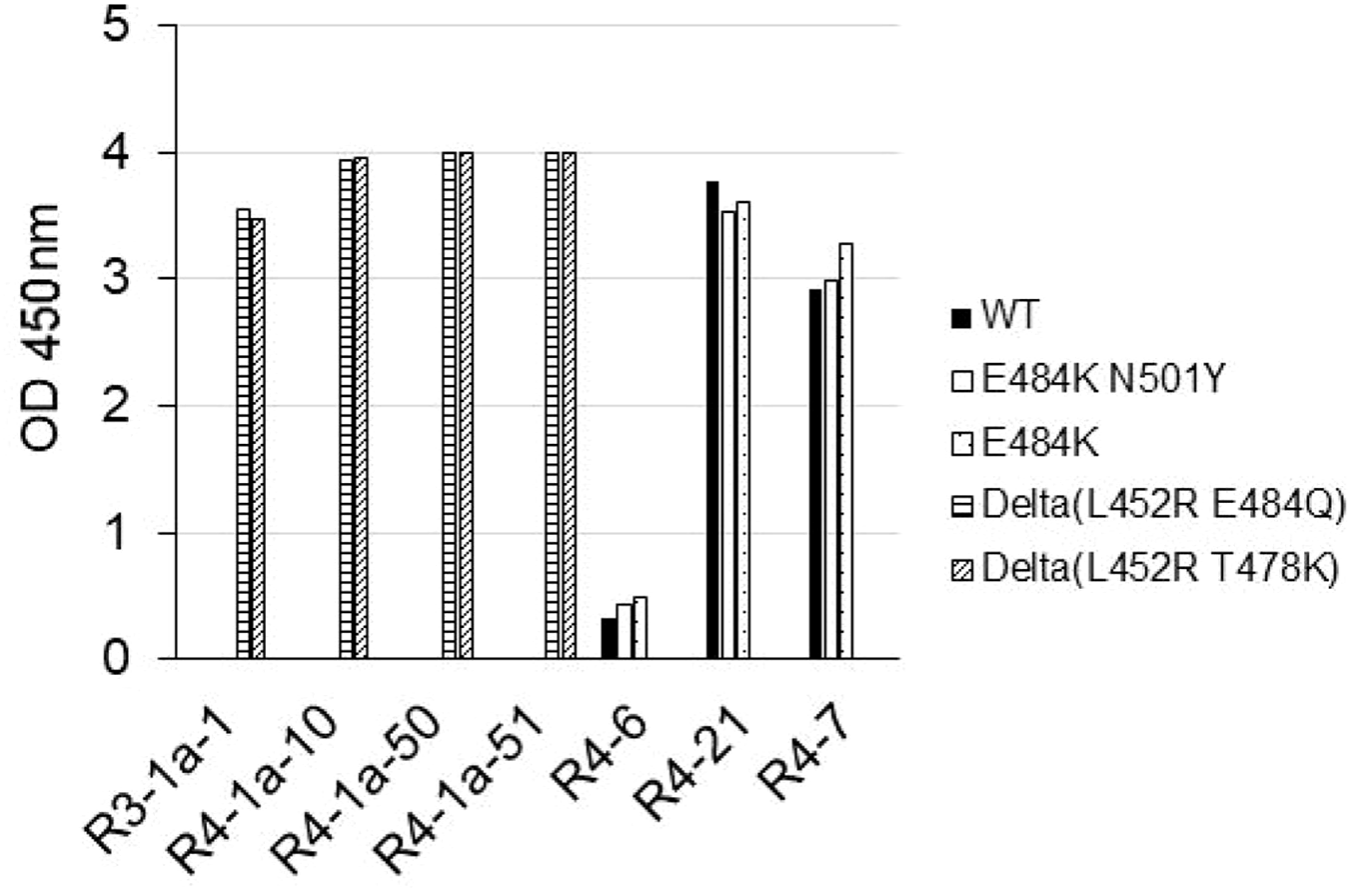
Characterization of phagemid clones of Fab fragments. Phagemid clone of Fab fragments against wildtype (WT), and E484K, E484K/N501Y, L452R/E484Q, L452R/T478K variants of spike RBD were evaluated by ELISA. Seven clones with capacity of development were selected and shown.

Using ELISA, the phagemid clones encoding Fab fragments targeted against the wildtype (WT) and the SARS-CoV-2 spike RBD variants E484K, E484K/N501Y, L452R/E484Q, and L452R/T478K were evaluated. Seven mAb clones — R3-1a-1, R4-1a-10, R4-1a-50, R4-1a-51, R4-6, R4-7, and R4-21 — demonstrated potential for further development and were selected based on their performance. (**Figure 1**).

### Generation and purification of the selected monoclonal and bispecific antibodies

The selected mAb constructs (R3-1a-1, R4-21, R4-1a-10, and R4-1a-51) were analyzed by reducing SDS-PAGE, which showed the expected heavy- and light-chain bands for all selected mAbs. These electrophoretic profiles were consistent with the expected chain composition and apparent molecular weights of the produced antibodies (**Supplementary Figure 3**).

SEC-HPLC analysis was conducted to assess aggregation of the produced anti-SARS-CoV-2 R3-1a-1/R4-1a-10 scFv bsAb at concentrations ranging from 1 mg/mL to 40 mg/mL. The analysis confirmed that the purity of the prepared bsAbs exceeded 99% across all tested concentrations (**Supplementary Figure 4**). In addition, μCE-SDS analysis showed that 97.05% of the R3-1a-1/R4-1a-10 scFv bsAb was present as the intact IgG species under non-reducing conditions (**Supplementary Figure 5**).

### Binding activity of selected monoclonal antibodies against Omicron subvariants

**Figure 2:**
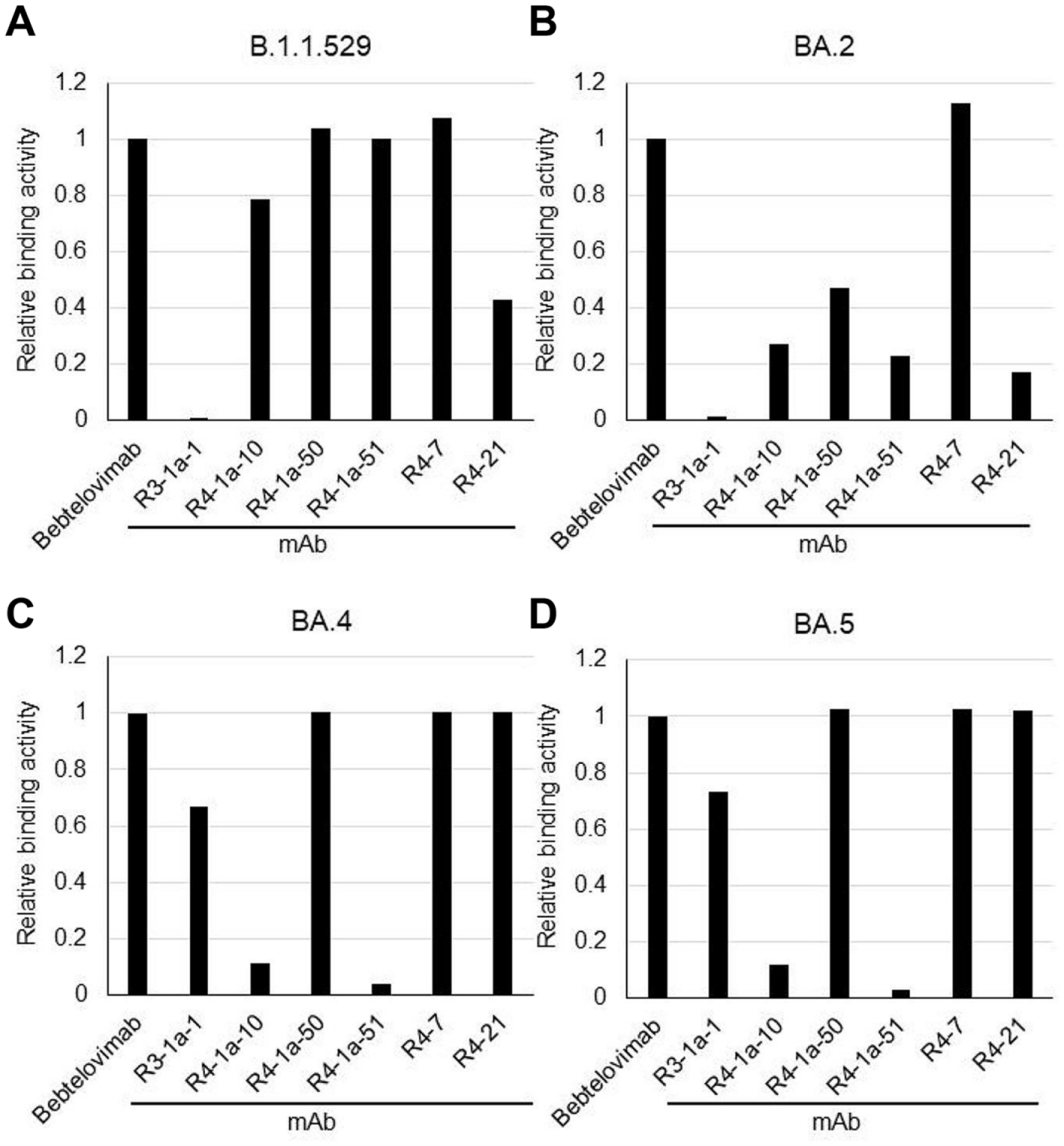
Relative binding activity of selected mAb clones against spike RBD proteins from Omicron subvariants. The binding activity of selected mAb clones (R4-6, R4-7, R3-1a-1, R4-21, R4-1a-10, R4-1a-50, and R4-1a-51) against recombinant spike RBD proteins from Omicron subvariants B.1.1.529 **(A)**, BA.2 **(B)**, BA.4 **(C)**, and BA.5 **(D)** was evaluated by direct ELISA. Relative binding activity was normalized to Bebtelovimab, which was set to 1 for each subvariant separately. The selected clones showed distinct reactivity patterns across the tested Omicron subvariants.

To sample representative antigenically distinct Omicron sublineages, binding analyses were performed against B.1.1.529, BA.2, BA.4, and BA.5 (3, 5, 7, 20). The binding capacity of the selected mAb clones (R4-7, R3-1a-1, R4-21, R4-1a-10, R4-1a-50, R4-1a-51) to the spike RBD protein of Omicron subvariants B.1.1.529, BA.2, BA.4, and BA.5 was analyzed, with Bebtelovimab serving as the control. The elected mAbs showed varied binding activity against different Omicron subvariants. Clone R3-1a-1 and R4–21 had high binding activity against BA.4 and BA.5 but relatively lower against B.1.1.529 and BA.2 variants. Clone R4-1a-10 and R4-1a-51 had better binding activity against B1.1.529 and BA.2, but relatively lower binding activity against BA.4 and BA.5. Additionally, clone R4-1a-50 and R4–7 showed strong binding ability against all the Omicron subvariants (**Figure 2**). These results demonstrated complementary binding patterns among the selected mAbs across the tested Omicron subvariants, with R3-1a-1 and R4–21 showing relatively stronger reactivity to BA.4/BA.5, whereas R4-1a-10 and R4-1a-51 showed relatively stronger reactivity to B.1.1.529/BA.2.

### Kinetic properties of selected monoclonal antibodies against Omicron spike proteins

Rapid kinetic properties of the mAb clones R3-1a-1, R4-21, R4-1a-10, and R4-1a-51 against Omicron subvariants B1.1.529, BA.2, BA.4, and BA.5 were tested, demonstrated by the dissociation constant (K_D_), with lower K_D_ values indicating a more favorable kinetic profile. Overall, R4-1a-10 demonstrates the highest affinity across the four subvariants, especially for B1.1.529, while R4-1a-51 shows the most variable affinity, with significantly weaker binding to BA.4 and BA.5 compared to the other clones and subvariants (**Table 1**). Together with the complementary binding patterns observed in **Figure 2**, these data provided the basis for selecting parental mAbs for bsAb construction. Because the tested RBD proteins and Omicron subvariant spike/RBD proteins differ by defined amino acid substitutions (**Supplementary Table 1**), including residues previously implicated in antibody escape such as L452, F486, and Q493 (3), the variant-dependent binding and kinetic changes provide indirect information on mutation-sensitive RBD recognition patterns. However, these data should not be interpreted as definitive epitope mapping.

**Table 1:** Rapid kinetic properties of mAb clones R3-1a-1, R4-21, R4-1a-10 and R4-1a-51 against Omicron subvariants B1.1.529, BA.2, BA.4 and BA.5.

mAb clone	K_D_ (M)			
	B1.1.529	BA.2	BA.4	BA.5
R4-1a-10	5.71E-10	3.23E-09	6.86E-09	7.86E-09
R4-1a-51	3.49E-09	1.03E-08	1.11E-05	2.53E-04
R3-1a-1	N.D.	4.36E-09	1.03E-08	1.25E-08
R4-21	2.58E-09	2.32E-09	1.62E-09	2.63E-09

### Binding activity of the bispecific antibodies against Omicron subvariants

**Figure 3:**
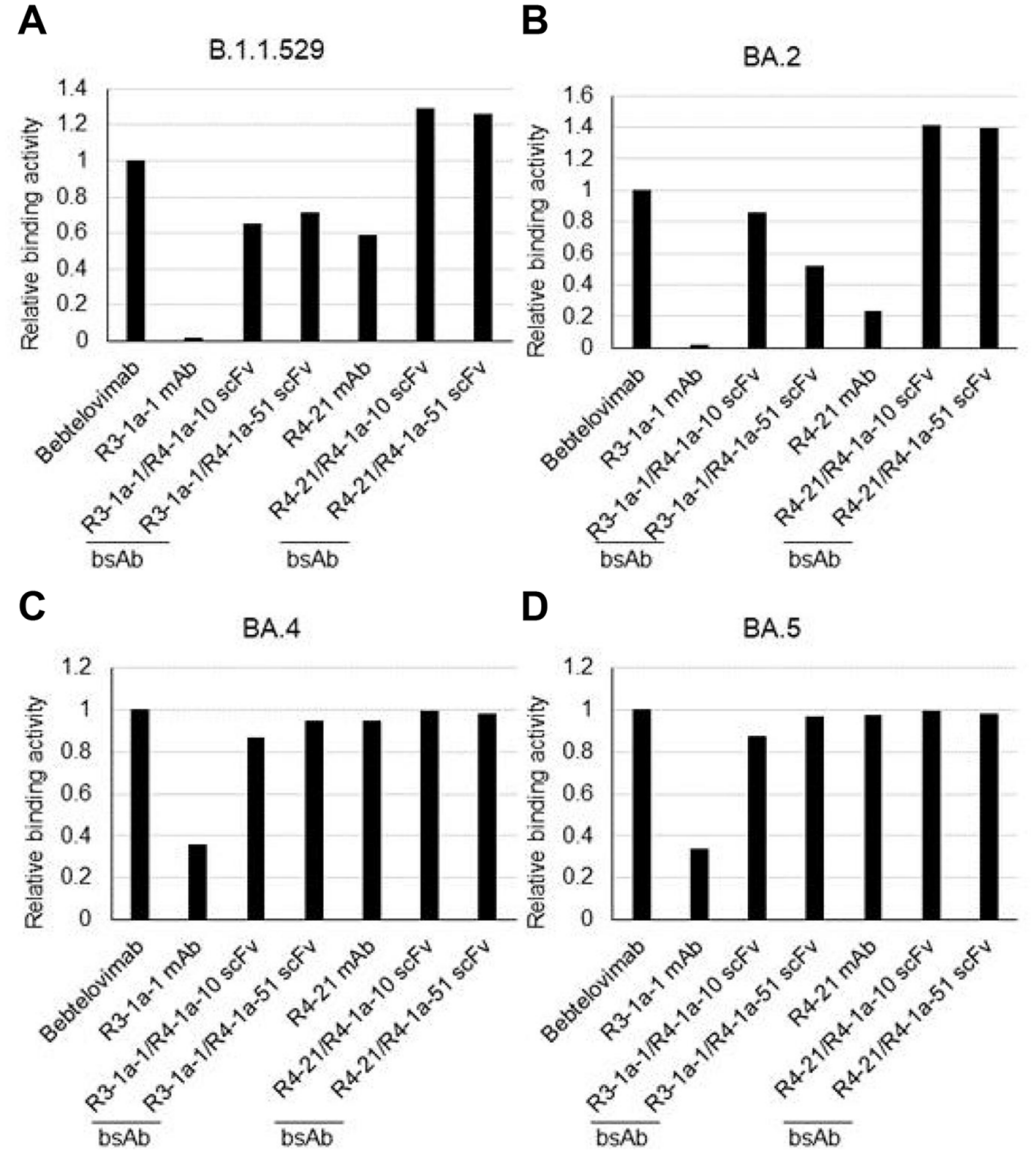
Relative binding activity of bsAbs and selected parental mAbs against spike RBD proteins from Omicron subvariants. The binding activity of bsAbs (R3-1a-1/R4-1a-10, R3-1a-1/R4-1a-51, R4-21/R4-1a-10, and R4-21/R4-1a-51), selected parental mAbs (R3-1a-1 and R4-21), and the control antibody Bebtelovimab against recombinant spike RBD proteins from Omicron subvariants B.1.1.529 **(A)**, BA.2 **(B)**, BA.4 **(C)**, and BA.5 **(D)** was evaluated by direct ELISA. Relative binding activity was normalized to Bebtelovimab, which was set to 1 for each subvariant separately. The selected bsAbs showed comparable binding activity in BA.4 and BA.5 and variable binding activity across the other tested subvariants.

Based on the complementary binding patterns and kinetic profiles of the parental mAbs across the tested Omicron subvariants, four bsAbs were designed in the IgG1-scFv format, generating potent antibodies R3-1a-1/R4-1a-10, R3-1a-1/R4-1a-51, R4-21/R4-1a-10 and R4-21/R4-1a-51 scFv. The binding activity of these bsAbs, together with mAbs R3-1a-1 and R4–21 and the control antibody Bebtelovimab, against spike RBD proteins from Omicron subvariants B.1.1.529, BA.2, BA.4, and BA.5 was then evaluated. As a result, the selected bsAbs demonstrated, in BA.4 and BA.5 subvariants, a binding activity higher than that of the R3-1a-1 mAb, with comparable binding activity between each other. In B1.1.529 and BA.2 subvariants, all the selected bsAbs also showed better binding activities than mAbs R3-1a-1 and R4-21 (**Figure 3**).

### Neutralizing efficacy of the bispecific antibodies

**Figure 4:**
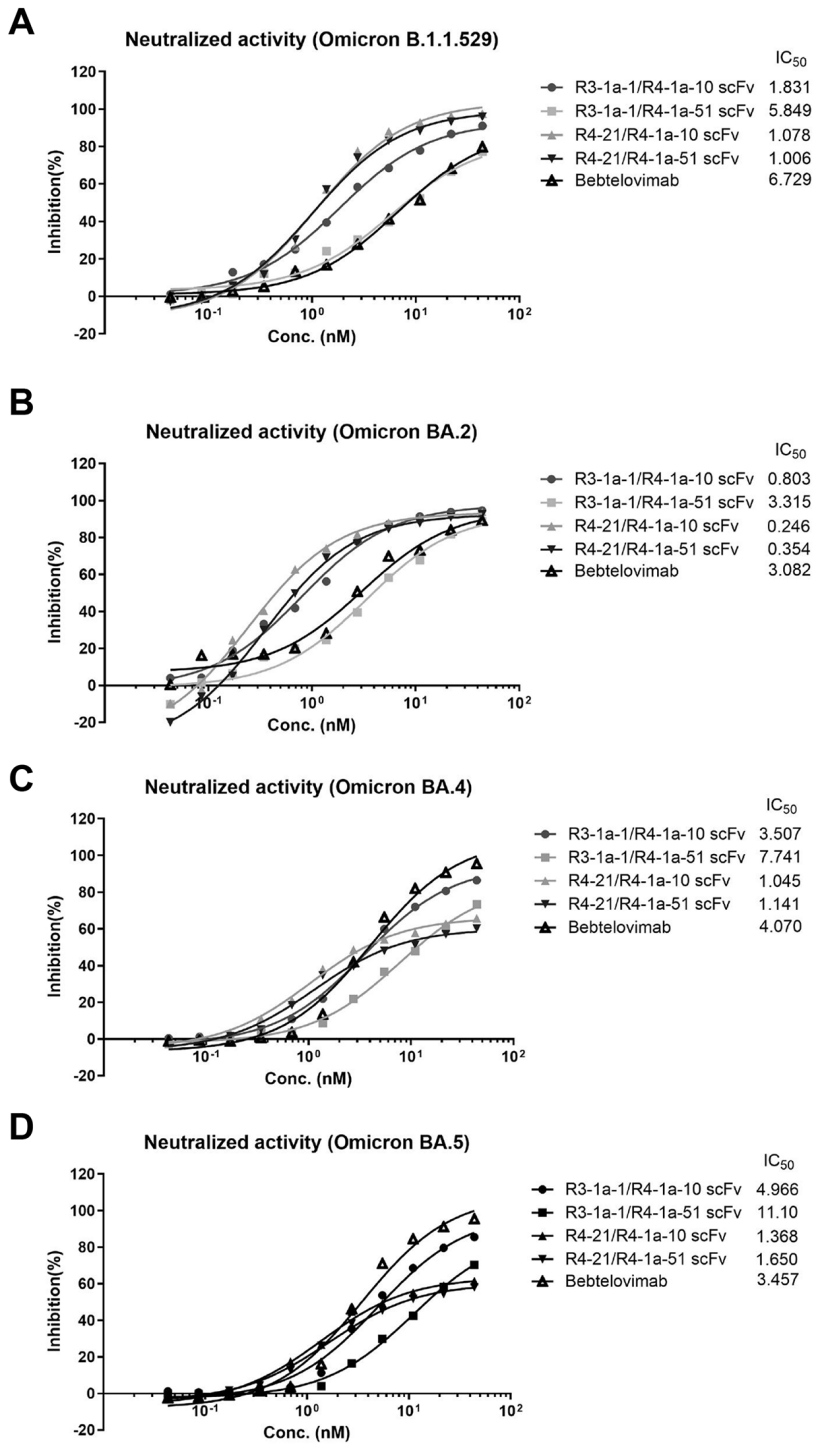
Neutralizing efficacy of bsAbs in the ACE2-blocking assay against Omicron subvariants. **(A)** Omicron B.1.1.529. **(B)** Omicron BA.2. **(C)** Omicron BA.4. **(D)** Omicron BA.5. Neutralizing activity was evaluated by competitive ELISA measuring inhibition of recombinant spike protein binding to hACE2. The inhibition curves are shown as percentage inhibition versus antibody concentration, and IC50 values represent the antibody concentration required to inhibit 50% of spike–ACE2 binding. Compared with Bebtelovimab, R4-21/R4-1a-10 and R4-21/R4-1a-51 scFv showed lower IC50 values against all tested Omicron subvariants. In addition, R3-1a-1/R4-1a-10 showed lower IC50 values against B.1.1.529, BA.2, and BA.4 than Bebtelovimab.

The bsAbs’ neutralization efficacy compared against Omicron subvariants B1.1.529, BA.2, BA.4, and BA.5 binding to hACE2 receptor proteins were tested. Compared with Bebtelovimab, R4-21/R4-1a-10 and R4-21/R4-1a-51 scFv showed lower IC50 against all Omicron subvariants. In addition, R3-1a-1/R4-1a-10 showed lower IC50 against B1.1.529, BA.2, and BA.4 than Bebtelovimab. (**Figure 4**).

### Comparison of bsAbs’ neutralization efficacy with cocktails of mAbs against the SARS-CoV-2 pseudovirus

**Figure 5:**
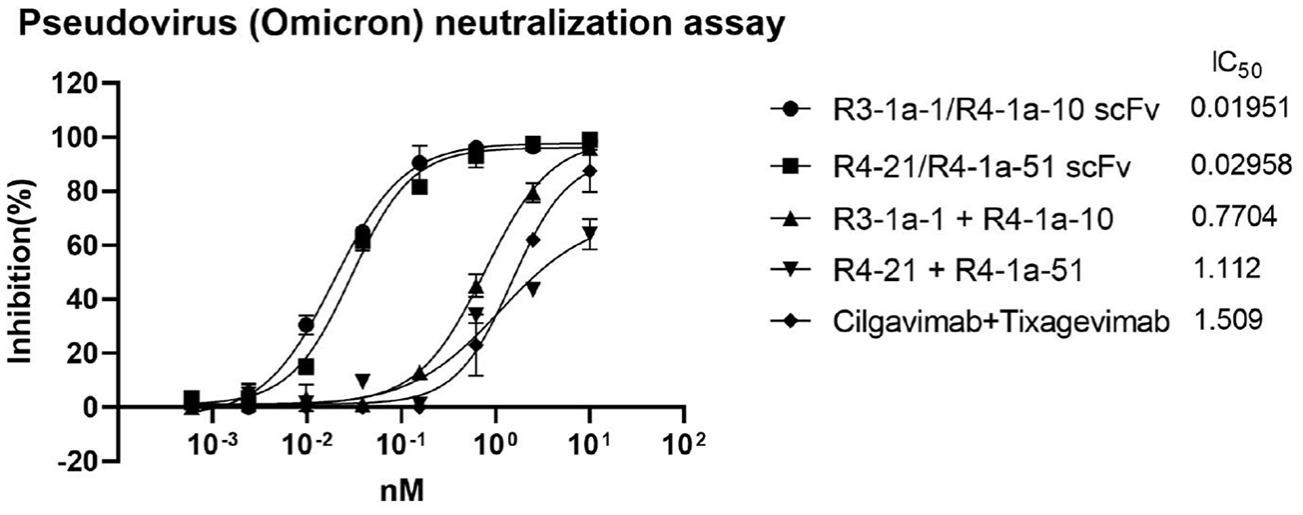
Comparison of bsAb neutralization efficacy with mAb cocktails in a lentiviral SARS-CoV-2 Omicron (B.1.1.529) pseudovirus assay. Neutralization of pseudoviral entry into 293T-hACE2 cells was quantified by measuring firefly luciferase activity. The percentage of neutralization was calculated relative to virus-only control wells. IC50 values were derived from dose-response curves fitted by variable-slope nonlinear regression in GraphPad Prism. The bsAbs R3-1a-1/R4-1a-10 scFv and R4-21/R4-1a-51 scFv were compared with the corresponding parental mAb cocktails (R3-1a-1 + R4-1a-10 and R4-21 + R4-1a-51) and the clinical benchmark cocktail Cilgavimab + Tixagevimab. For cocktail comparisons, antibodies were combined at a fixed 1:1 molar ratio and tested in parallel with bsAbs under the same assay conditions, with total antibody concentrations matched at each dilution point. Lower IC50 values indicate stronger neutralizing activity. Data points represent biological duplicate measurements, and error bars indicate SEM.

The neutralization efficiency of the selected bsAbs comparing with mAb cocktails was analyzed in a SARS-CoV-2 Omicron (B.1.1.529) pseudovirus model. Lower IC_50_ of the R3-1-a-1/R4-1a-10 and R4-21/R4-1a-51 scFv are shown, indicating stronger neutralizing efficacy than the mAb cocktails and the control (Cilgavimab + Tixagevimab) (**Figure 5**). These results supported that these bsAbs may represent the useful therapeutic agents for authentic virus to enable a neutralized response to Omicron subvariants.

The neutralization efficacy of bsAbs was compared with that of mAb cocktails, each comprising two mAbs, against SARS-CoV-2 pseudovirus (WT, Alpha, and Delta strains) targeting hACE2 receptor proteins. Imdevimab + Casirivimab was used as the commercial comparator in these non-Omicron pseudovirus assays because REGEN-COV was an established authorized antibody combination for earlier SARS-CoV-2 strains, whereas its activity was markedly reduced against Omicron and therefore it was not selected as the primary comparator for the Omicron-focused assays (21, 22). Lower IC50 values were observed for the R3-1a-1/R4-1a-10 and R4-21/R4-1a-51 scFv constructs, demonstrating their superior neutralizing efficacy compared to both the mAb cocktails and controls across strains other than Omicron (**Supplementary Figure 6**).

## Discussion

Two bsAbs, namely R3-1a-1/R4-1a-10 scFv and R4-21/R4-1a-51 scFv, showed strong spike-protein binding and pseudoviral neutralizing activity against the tested Omicron subvariants, as well as against WT, Alpha, and Delta strains evaluated in this study. The inclusion of B.1.1.529, BA.2, BA.4, and BA.5 was intended to sample early and later Omicron lineages with distinct immune-evasion profiles rather than to represent the full spectrum of current or future Omicron evolution. Taken together, these findings support the further preclinical development of these bsAbs.

The evolutionary rate of SARS-CoV-2 has been estimated to range from 9.9×10^−4^ and 2.2×10^−3^ mutations per site per year, which is comparable to that of other RNA viruses undergoing active transmission (23). This high mutation rate has led to the emergence of multiple SARS-CoV-2 variants and continues to pose a major challenge for COVID-19 control. Since the outbreak of COVID-19, nine anti-SARS-CoV-2 prophylactic and/or therapeutic antibody-based drugs have been authorized by the US Food and Drug Administration (FDA) and/or European Agency of Medicines (EMA) (24). However, the antiviral efficacy of several antibody-based drugs has gradually declined as new variants have emerged (24–27). At the time of writing, Cilgavimab plus Tixagevimab combination therapy and Bebtelovimab monotherapy represented the remaining antibody-based options for the treatment of COVID-19, including infections caused by Omicron subvariants (28, 29). Accordingly, Cilgavimab plus Tixagevimab was selected as a clinically relevant benchmark cocktail for pseudovirus neutralization comparisons, whereas Bebtelovimab was used as the single-antibody comparator in the Omicron binding and ACE2-blocking assays. By contrast, Imdevimab + Casirivimab was used as the commercial comparator in the WT, Alpha, and Delta pseudovirus assays because REGEN-COV was a clinically established comparator for pre-Omicron SARS-CoV-2 strains but showed markedly reduced neutralizing activity against Omicron (21, 22). Nonetheless, both antibody cocktails and single mAb may continue to face challenges as viral mutation accumulates. Therefore, the development of agents with a broader neutralizing activity against SARS-CoV-2 variants is a worthwhile goal. In this study, the R4-21/R4-1a-51 scFv bsAb showed higher affinity and lower IC50 against all the tested Omicron subvariants in the spike protein neutralization assay compared with Bebtelovimab. Similarly, R3-1a-1/R4-1a-10 showed lower IC50 against BA.1.1.529, BA.2, and BA.4, although not against BA.5.

Various antibody formats have been explored for antiviral therapy, including conventional mAbs, shark variable new antigen receptor (VNAR) antibodies, single-chain variable fragments (scFv), and bsAbs (30–32). Although VNARs and scFvs offer advantages in epitope accessibility and genetic manipulability, their relatively small molecular size places them below the glomerular filtration cutoff, resulting in substantially shorter clinical half-lives (33). By contrast, mAbs remain a powerful platform for combating a wide range of pathogens because they can specifically target viral antigens and reduce viral burden, hospitalization, and mortality (34, 35). However, a significant challenge arises due to the high mutation rates observed in RNA viruses. This rapid mutation often leads to alterations in the mAb’s targeted recognition site. Consequently, mAbs designed against the original virus strain may no longer effectively combat mutated virus variants, ultimately diminishing their therapeutic efficacy. This phenomenon has been exemplified in the case of mAbs developed against SARS-CoV-2. To address this challenge, combination therapies involving mAbs that target different receptors or epitopes have been explored. Such an approach can enhance the overall treatment efficacy and help overcome issues related to drug resistance (36). The present study extends this concept by using a non-conventional bsAb format capable of simultaneously engaging multiple receptors or epitopes. Prior SARS-CoV-2 bsAb studies have shown that format selection, epitope pairing, and mechanistic design can substantially influence neutralization breadth and potency (12, 16–18). Here, phage-display screening served as an accessible and cost-effective approach to identify fully human parental antibodies, rather than as a novel technology in itself. The main contribution of this work is the downstream engineering and functional evaluation of selected parental antibodies as bsAb candidates, which showed improved *in vitro* binding and neutralizing activity across the tested Omicron panel compared with selected parental antibodies, antibody cocktails, and commercial controls.

In the present study, the improved performance of selected bsAbs in the binding and neutralization assays may reflect the functional advantage of combining parental antibodies with complementary reactivity profiles across the tested subvariants. However, because epitope binning, competition studies, and structural characterization were not performed, the precise epitope relationships and spatial basis of this apparent functional advantage remain to be determined. Notably, in the pseudovirus assay used here, the selected bsAbs showed stronger neutralizing activity than the Cilgavimab plus Tixagevimab cocktail.

This interpretation is further supported by the observation that the parental mAbs R3-1a-1 and R4-1a-10 exhibited distinct recognition profiles across the tested Omicron subvariants, whereas the bsAb generated by combining these two binding arms showed broader and stronger activity in the corresponding assays. More generally, the improved activity of certain bsAbs relative to some parental antibodies and mAb cocktails may indicate a functional benefit derived from combining antibodies with complementary binding and kinetic profiles. Although an allosteric or geometry-related effect is one possible explanation (37, 38), the current study did not include epitope binning, mutational mapping, or structural analyses. Therefore, any mechanistic interpretation regarding dual-epitope engagement, spatial complementarity, or allosteric enhancement should be considered preliminary.

### Strength and limitations

This study identified and characterized several fully human anti-SARS-CoV-2 antibody leads and demonstrated that selected bsAbs showed improved binding and neutralizing activity against the tested Omicron subvariants when compared with selected parental antibodies, mAb cocktails, and commercial controls in *in vitro* assays. The integration of phage-display screening, mAb reformatting, kinetic analysis, receptor-blocking assays, pseudovirus neutralization, and biochemical characterization provides a useful framework for evaluating candidate bsAbs against rapidly evolving SARS-CoV-2 variants.

Nevertheless, several limitations should be acknowledged. **First**, the mechanistic basis underlying bsAb pairing and performance remains incompletely defined, because epitope binning, competition assays with reference antibodies of known RBD epitope classes, systematic mutational mapping, and structural characterization were not performed in the present study. Although the differential binding and kinetic profiles against RBD mutants and Omicron subvariant spike/RBD proteins, together with the amino acid substitution information summarized in **Supplementary Table 1**, provide indirect information on mutation-sensitive RBD recognition patterns, these data do not define the precise binding epitopes or spatial geometry of the parental antibodies. Therefore, the precise spatial relationship between the two binding arms and the extent of true epitope complementarity remain to be determined. **Second**, the current study evaluated only the tested Omicron subvariants and did not include resistance/escape experiments, which will be important for assessing the breadth and durability of bsAb activity under continued viral evolution. **Third**, the *in vitro* assays used here, including ACE2-blocking and pseudovirus assays, do not fully recapitulate authentic-virus infection or *in vivo* conditions. **Finally**, long-term developability issues, including thermostability, pharmacokinetics, and immunogenicity, were not addressed and will require further investigation.

## Conclusions

This study identified two lead bispecific antibodies, R3-1a-1/R4-1a-10 scFv and R4-21/R4-1a-51 scFv, that showed enhanced *in vitro* binding and neutralizing activity across the tested Omicron subvariants compared with selected parental antibodies, antibody cocktails, and commercial controls. Notably, these bsAbs also retained neutralizing activity in the additional pseudovirus assays using WT, Alpha, and Delta strains evaluated in this study. Together, these findings support bispecific antibody engineering as a promising strategy for improving antibody performance against antigenically diverse SARS-CoV-2 variants and provide a basis for further preclinical development of these lead constructs.

## Data Availability

The original contributions presented in the study are included in the article/**Supplementary Material**. Further inquiries can be directed to the corresponding author.

## Supplementary material

Supplementary Figure 1 Structural representation of the symmetric format of anti-SARS-CoV-2 bsAb.

Supplementary Figure 2 Phage ELISA-based enrichment of SARS-CoV-2 spike RBD-binding phage populations after biopanning with the Omni-Mab library. **(A)** Polyclonal phage pools obtained after four rounds of panning against Omicron-related RBD variants (WT, E484K, and E484K/N501Y) were evaluated by phage ELISA. **(B)** Polyclonal phage pools obtained after four rounds of panning against Delta-related RBD variants (WT, L452R/E484Q, and L452R/T478K) were evaluated by phage ELISA. For panels **(A, B)**, bound phages were detected with an HRP-conjugated anti-M13 antibody, and binding activity is shown as OD450. **(C)** Enrichment yield (fold change) of recovered phage outputs after four rounds of biopanning against the indicated SARS-CoV-2 RBD variants. Data are presented as mean ± SEM. Statistical significance versus control was analyzed using a two-tailed unpaired Student’s *t*-test; *****P* < 0.0001.

Supplementary Figure 3 Reducing SDS-PAGE analysis of selected mAb constructs (R3-1a-1, R4-21, R4-1a-10, and R4-1a-51). All selected antibodies showed the expected heavy-chain and light-chain bands under the reducing conditions.

Supplementary Figure 4 SEC-HPLC analysis of protein aggregation in anti-SARS-CoV-2 R3-1a-1/R4-1a-10 scFv bsAb at various concentrations. The main peak values, indicative of the antibody’s monomeric form, are summarized in the accompanying table. The purity of selected bsAb was higher than 99% in each concentration.

Supplementary Figure 5 μCE-SDS analysis of the anti-SARS-CoV-2 R3-1a-1/R4-1a-10 scFv bispecific antibody. **(A)** Non-reducing μCE-SDS electropherogram of the purified R3-1a-1/R4-1a-10 scFv bsAb, showing that the intact IgG species accounted for 97.05% of the total detected protein. **(B)** Reducing μCE-SDS electropherogram of the same bsAb, showing the expected heavy-chain (HC) and light-chain (LC) species, which accounted for 76.98% and 22.03% of the total signal, respectively. μCE-SDS was performed using the Protein Clear HR Assay on a LabChip GXII instrument under non-reducing and reducing conditions.

Supplementary Figure 6 Comparison of the bsAb neutralization efficacy with mAbs cocktails against the SARS-CoV-2 WT, Alpha, and Delta pseudoviruses. **(A)** WT pseudovirus neutralization assay. **(B)** Alpha pseudovirus neutralization assay. **(C)** Delta pseudovirus neutralization assay. Neutralization of pseudoviral entry into 293T-hACE2 cells was quantified using a luciferase readout, and IC50 values were calculated by nonlinear regression. The bsAbs R3-1a-1/R4-1a-10 scFv and R4-21/R4-1a-51 scFv were compared with the corresponding parental mAb cocktails and with the commercial comparator Imdevimab + Casirivimab, which was included as a benchmark antibody cocktail for earlier non-Omicron SARS-CoV-2 strains. For cocktail comparisons, antibodies were combined at a fixed 1:1 molar ratio and tested in parallel with bsAbs under the same assay conditions. Lower IC50 values indicate stronger neutralizing activity. Data points represent duplicate measurements, and error bars indicate SEM.

## References

[1] Fan Y Li X Zhang L Wan S Zhang L Zhou F . SARS-CoV-2 Omicron variant: recent progress and future perspectives. Signal Transd Targ Ther. (2022) 7:141. doi: 10.1038/s41392-022-00997-x 35484110 PMC9047469

[2] Silva S Kohl A Pena L Pardee K . Recent insights into SARS-CoV-2 omicron variant. Rev Med Virol. (2023) 33:e2373. doi: 10.1002/rmv.2373 35662313 PMC9347414

[3] Cao Y Yisimayi A Jian F Song W Xiao T Wang L . BA.2.12.1, BA.4 and BA.5 escape antibodies elicited by Omicron infection. Nature. (2022) 608:593–602. doi: 10.1038/s41586-022-04980-y 35714668 PMC9385493

[4] Liu L Iketani S Guo Y Chan JF Wang M Liu L . Striking antibody evasion manifested by the Omicron variant of SARS-CoV-2. Nature. (2022) 602:676–81. doi: 10.1038/s41586-021-04388-0 35016198

[5] Xia S Wang L Zhu Y Lu L Jiang S . Origin, virological features, immune evasion and intervention of SARS-CoV-2 Omicron sublineages. Signal Transd Targ Ther. (2022) 7:241. doi: 10.1038/s41392-022-01105-9 35853878 PMC9295084

[6] Alam MS . Insight into SARS-CoV-2 Omicron variant immune escape possibility and variant independent potential therapeutic opportunities. Heliyon. (2023) 9:e13285. doi: 10.1016/j.heliyon.2023.e13285 36744070 PMC9886571

[7] Junker D Becker M Wagner TR Kaiser PD Maier S Grimm TM . Antibody binding and angiotensin-converting enzyme 2 binding inhibition is significantly reduced for both the BA.1 and BA.2 Omicron variants. Clin Infect Dis. (2023) 76:e240–9. doi: 10.1093/cid/ciac498 35717657 PMC9384292

[8] Shang L Cao B . Adapted vaccine strategy: facing the persistent challenges of COVID-19. Lancet Infect Dis. (2023) 23:984–5. doi: 10.1016/s1473-3099(23)00370-5 37348518 PMC10278996

[9] Cao Y Jian F Zhang Z Yisimayi A Hao X Bao L . Rational identification of potent and broad sarbecovirus-neutralizing antibody cocktails from SARS convalescents. Cell Rep. (2022) 41:111845. doi: 10.1016/j.celrep.2022.111845 36493787 PMC9712074

[10] Yang Y Du L . Neutralizing antibodies and their cocktails against SARS-CoV-2 Omicron and other circulating variants. Cell Mol Immunol. (2022) 19:962–4. doi: 10.1038/s41423-022-00890-1 35750901 PMC9243717

[11] Guo Y Zhang G Yang Q Xie X Lu Y Cheng X . Discovery and characterization of potent pan-variant SARS-CoV-2 neutralizing antibodies from individuals with Omicron breakthrough infection. Nat Commun. (2023) 14:3537. doi: 10.1038/s41467-023-39267-x 37322000 PMC10267556

[12] Yang H Chen Y Jiang D Feng X Xu Y Wei J . Preclinical evaluation of ISH0339, a tetravalent broadly neutralizing bispecific antibody against SARS-CoV-2 with long-term protection. Antib Ther. (2023) 6:97–107. doi: 10.1093/abt/tbad003 37077474 PMC10108555

[13] Wang Y Hao A Ji P Ma Y Zhang Z Chen J . A bispecific antibody exhibits broad neutralization against SARS-CoV-2 Omicron variants XBB.1.16, BQ.1.1 and sarbecoviruses. Nat Commun. (2024) 15:5127. doi: 10.1038/s41467-024-49096-1 38879565 PMC11180174

[14] Li C Zhan W Yang Z Tu C Hu G Zhang X . Broad neutralization of SARS-CoV-2 variants by an inhalable bispecific single-domain antibody. Cell. (2022) 185:1389–1401.e1318. doi: 10.1016/j.cell.2022.03.009 35344711 PMC8907017

[15] Yuan M Chen X Zhu Y Dong X Liu Y Qian Z . A bispecific antibody targeting RBD and S2 potently neutralizes SARS-CoV-2 Omicron and other variants of concern. J Virol. (2022) 96:e0077522. doi: 10.1128/jvi.00775-22 35916510 PMC9400488

[16] Kim JW Kim HJ Heo K Lee Y Jang HJ Lee HY . A novel bispecific antibody dual-targeting approach for enhanced neutralization against fast-evolving SARS-CoV-2 variants. Front Immunol. (2023) 14:1271508. doi: 10.3389/fimmu.2023.1271508 37822941 PMC10562541

[17] Yuan M Zhu Y Liu G Wang Y Wang G Zhang G . An RBD bispecific antibody effectively neutralizes a SARS-CoV-2 Omicron variant. One Health Adv. (2023) 1:12. doi: 10.1186/s44280-023-00012-0 37521533 PMC10173222

[18] Li Z Zhang Z Rosen ST Feng M . Function and mechanism of bispecific antibodies targeting SARS-CoV-2. Cell Insight. (2024) 3:100150. doi: 10.1016/j.cellin.2024.100150 38374826 PMC10875118

[19] Gruell H Vanshylla K Korenkov M Tober-Lau P Zehner M Münn F . SARS-CoV-2 Omicron sublineages exhibit distinct antibody escape patterns. Cell Host Microbe. (2022) 30:1231–1241.e1236. doi: 10.1016/j.chom.2022.07.002 35921836 PMC9260412

[20] Wang Q Guo Y Iketani S Nair MS Li Z Mohri H . Antibody evasion by SARS-CoV-2 Omicron subvariants BA.2.12.1, BA.4 and BA.5. Nature. (2022) 608:603–8. doi: 10.1038/s41586-022-05053-w 35790190 PMC9385487

[21] U.S. Food and Drug Administration . Coronavirus (COVID-19) update: FDA authorizes monoclonal antibody for treatment of COVID-19 (2020). Available online at: https://www.fda.gov/news-events/press-announcements/coronavirus-covid-19-update-fda-authorizes-monoclonal-antibodies-treatment-covid-19 (Accessed April 16, 2026).

[22] Takashita E Kinoshita N Yamayoshi S Sakai-Tagawa Y Fujisaki S Ito M . Efficacy of antibodies and antiviral drugs against Covid-19 Omicron variant. N Engl J Med. (2022) 386:995–8. doi: 10.1056/nejmc2119407 35081300 PMC8809508

[23] Domingo E García-Crespo C Lobo-Vega R Perales C . Mutation rates, mutation frequencies, and proofreading-repair activities in RNA virus genetics. Viruses. (2021) 13:1882. doi: 10.3390/v13091882 34578463 PMC8473064

[24] Almagro JC Mellado-Sánchez G Pedraza-Escalona M Pérez-Tapia SM . Evolution of anti-SARS-CoV-2 therapeutic antibodies. Int J Mol Sci. (2022) 23:9763. doi: 10.3390/ijms23179763 36077159 PMC9456190

[25] U.S. Food and Drug Administration . Food and drug administration. Coronavirus (COVID-19) update: FDA revokes emergency use authorization for monoclonal antibody bamlanivimab. FDA; silver spring, MD, USA: 2021 (2021). Available online at: https://www.fda.gov/news-events/press-announcements/coronavirus-covid-19-update-fda-revokes-emergency-use-authorization-monoclonal-antibody-bamlanivimab (Accessed April 16, 2026).

[26] Takashita E Kinoshita N Yamayoshi S Sakai-Tagawa Y Fujisaki S Ito M . Efficacy of antiviral agents against the SARS-CoV-2 Omicron subvariant BA.2. N Engl J Med. (2022) 386:1475–7. doi: 10.1056/nejmc2201933 35263535 PMC8929374

[27] U.S. Food and Drug Administration . Coronavirus (COVID-19) update: FDA limits use of certain monoclonal antibodies to treat COVID-19 due to the omicron variant. Silver Spring, MD: FDA (2022) Available online at: https://www.fda.gov/news-events/press-announcements/coronavirus-covid-19-update-fda-limits-use-certain-monoclonal-antibodies-treat-covid-19-due-omicron (Accessed April 16, 2026).

[28] U.S. Food and Drug Administration . Fact sheet for healthcare providers: emergency use authorization for bebtelovimab. Silver Spring, MD: FDA (2022) Available online at: https://www.fda.gov/media/156152/download (Accessed April 16, 2026).

[29] U.S. Food and Drug Administration . Fact sheet for healthcare providers: emergency use authorization for EVUSHELD™ (tixagevimab co-packaged with cilgavimab). FDA: Silver Spring, MD (2022) Available online at: https://www.fda.gov/media/154701/download (Accessed April 16, 2026).

[30] Both L Banyard AC Van Dolleweerd C Wright E Ma JK Fooks AR . Monoclonal antibodies for prophylactic and therapeutic use against viral infections. Vaccine. (2013) 31:1553–9. doi: 10.1016/j.vaccine.2013.01.025 23370150 PMC7115371

[31] Reader RH Workman RG Maddison BC Gough KC . Advances in the production and batch reformatting of phage antibody libraries. Mol Biotechnol. (2019) 61:801–15. doi: 10.1007/s12033-019-00207-0 31468301 PMC6785589

[32] Pantaleo G Correia B Fenwick C Joo VS Perez L . Antibodies to combat viral infections: development strategies and progress. Nat Rev Drug Discov. (2022) 21:676–96. doi: 10.1038/s41573-022-00495-3 35725925 PMC9207876

[33] Asaadi Y Jouneghani FF Janani S Rahbarizadeh F . A comprehensive comparison between camelid nanobodies and single chain variable fragments. biomark Res. (2021) 9:87. doi: 10.1186/s40364-021-00332-6 34863296 PMC8642758

[34] Corti D Purcell LA Snell G Veesler D . Tackling COVID-19 with neutralizing monoclonal antibodies. Cell. (2021) 184:3086–108. doi: 10.1016/j.cell.2021.07.027 34087172 PMC8152891

[35] Tan J . Clonal wars: Monoclonal antibodies against infectious pathogens. DNA Cell Biol. (2022) 41:34–7. doi: 10.1089/dna.2021.0457 34941449 PMC8787689

[36] Scott AM Wolchok JD Old LJ . Antibody therapy of cancer. Nat Rev Cancer. (2012) 12:278–87. doi: 10.1038/nrc3236 22437872

[37] Al Qaraghuli MM Kubiak-Ossowska K Ferro VA Mulheran PA . Antibody-protein binding and conformational changes: identifying allosteric signalling pathways to engineer a better effector response. Sci Rep. (2020) 10:13696. doi: 10.1038/s41598-020-70680-0 32792612 PMC7426963

[38] Zhao J Nussinov R Ma B . The allosteric effect in antibody-antigen recognition. Methods Mol Biol. (2021) 2253:175–83. doi: 10.1007/978-1-0716-1154-8_11 33315224 PMC10802915

