# Supplementary material for: Development of bispecific antibodies with enhanced neutralization activity against tested SARS-CoV-2 Omicron subvariants
Source: Front Immunol. 2026 Jun 17;17:1793368. doi: 10.3389/fimmu.2026.1793368 (PMC13318592; doi:10.3389/fimmu.2026.1793368)
Supplement: Supplementary Figure 1 — Structural representation of the symmetric format of anti-SARS-CoV-2 bsAb. [file Presentation1.pptx]

## Slide 1
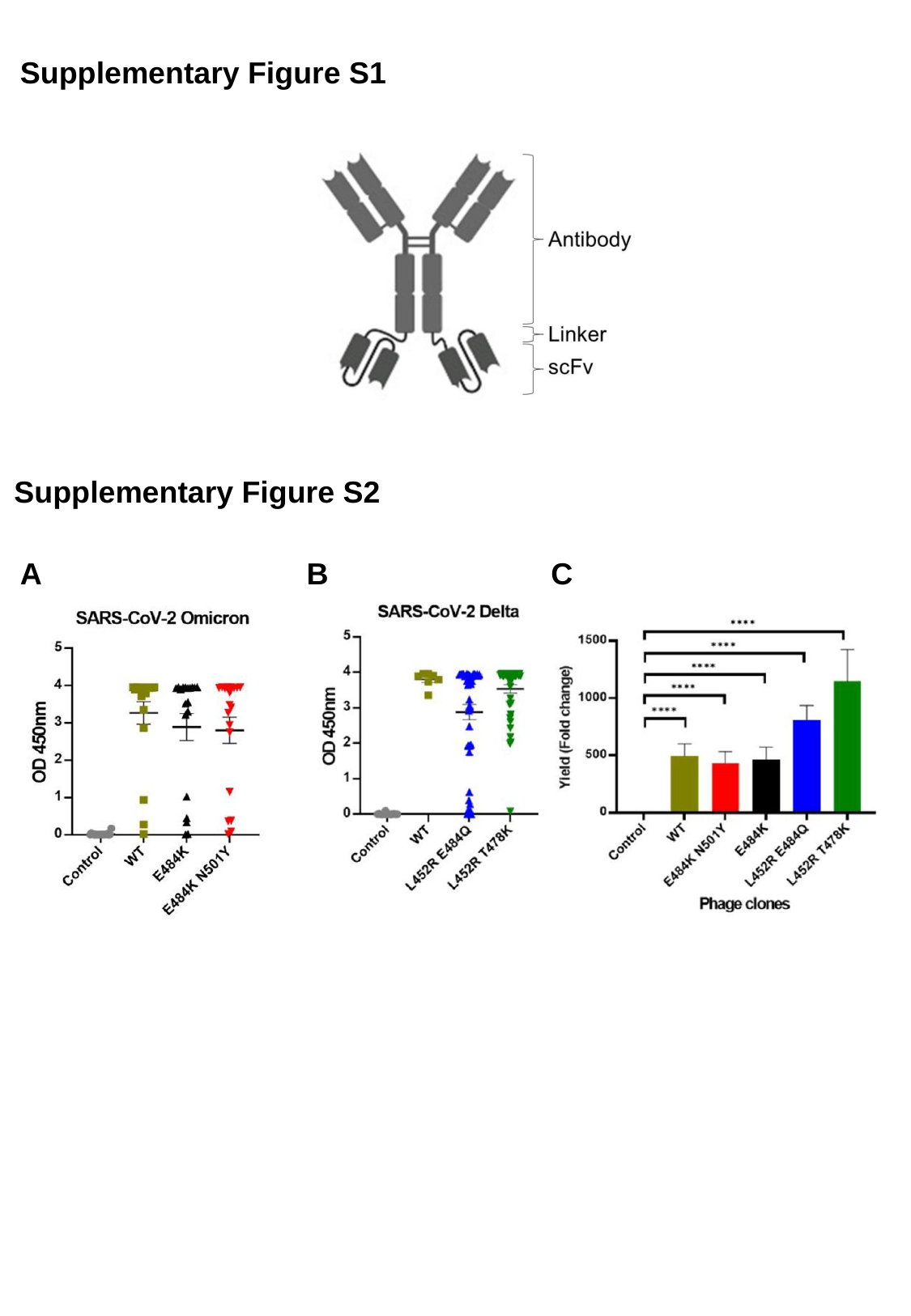

Supplementary Figure S1
Supplementary Figure S2
A
B
C

## Slide 2
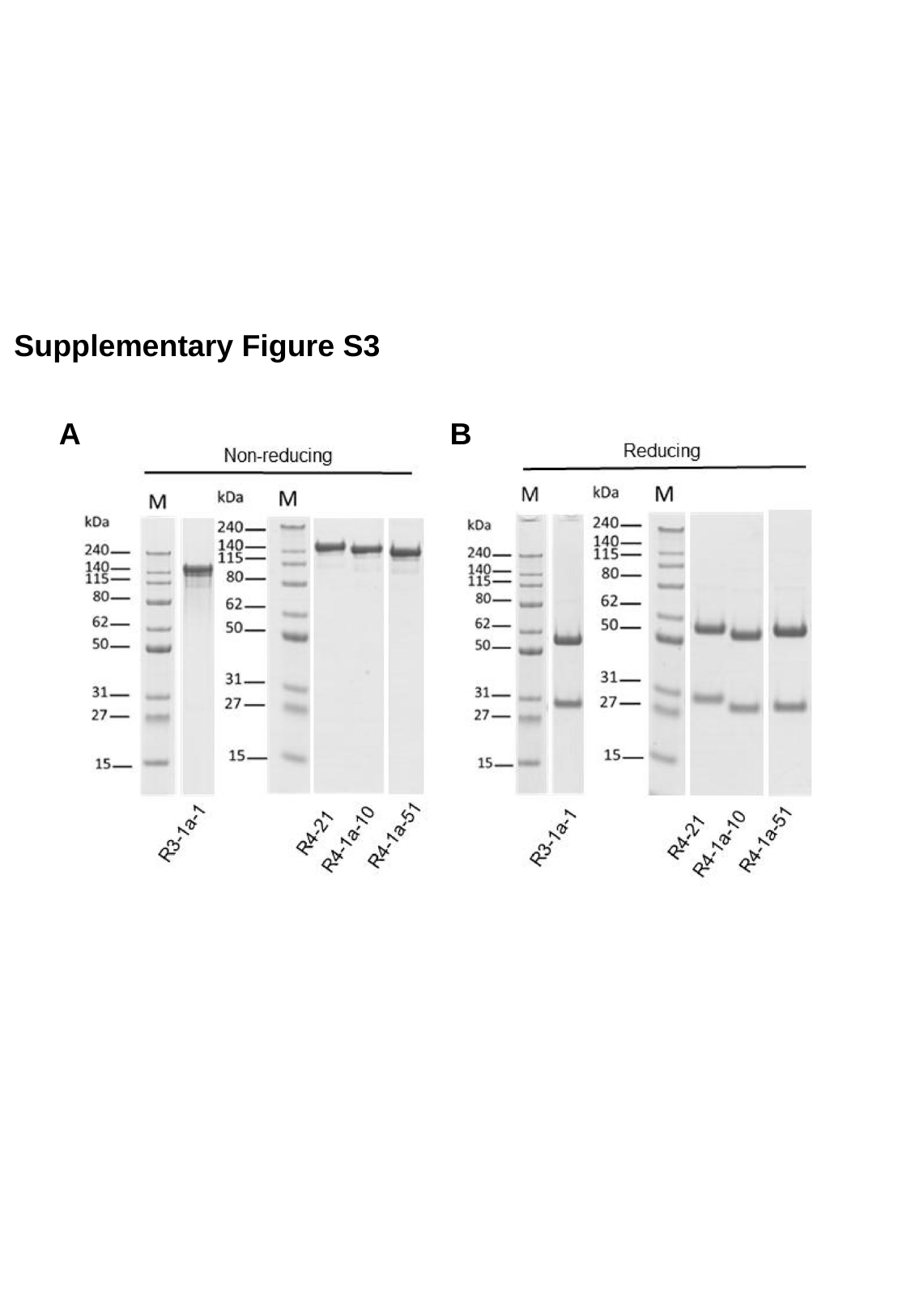

Supplementary Figure S3
A
B

## Slide 3
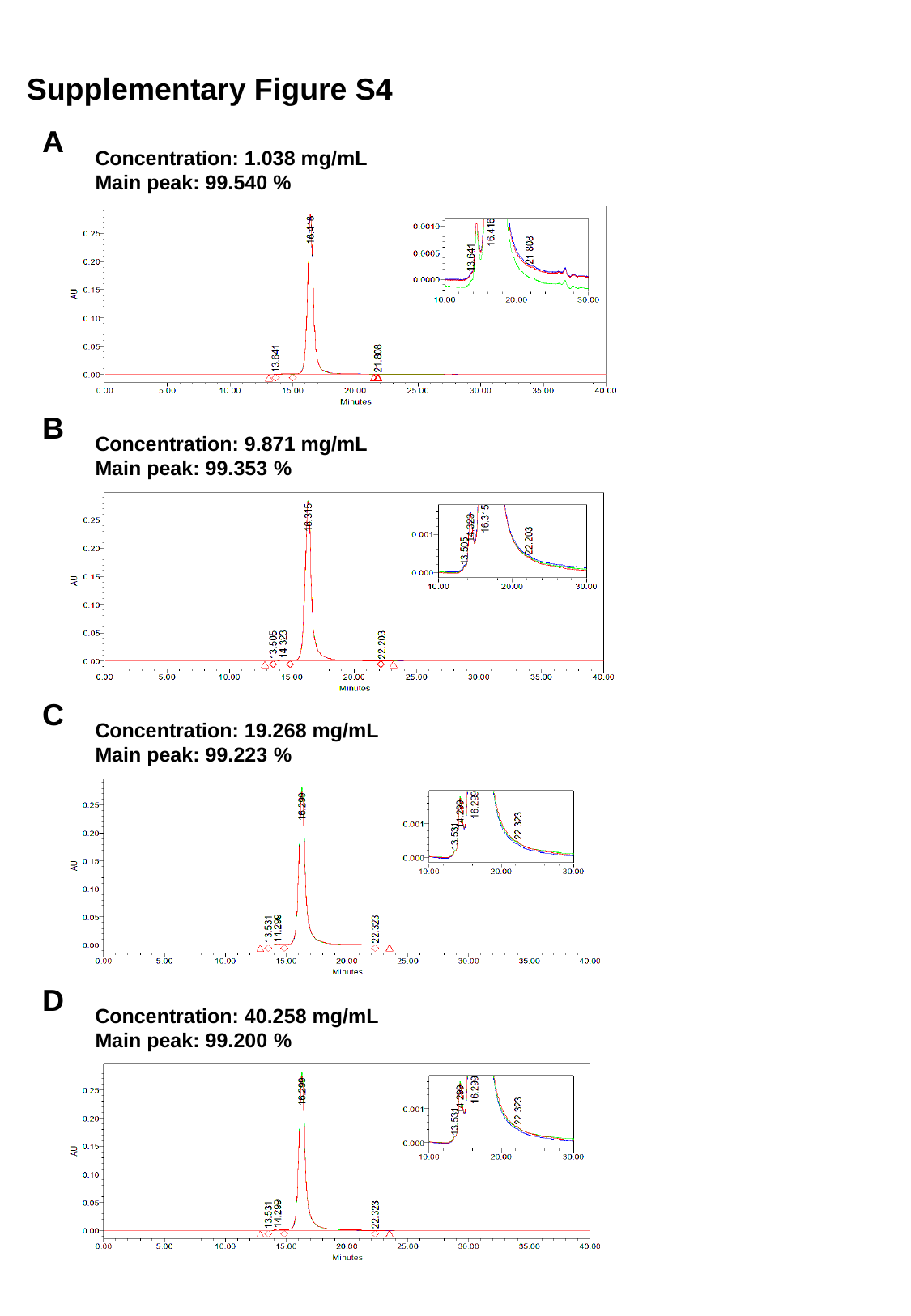

Supplementary Figure S4
A
Concentration: 1.038 mg/mL
Main peak: 99.540 %
B
Concentration: 9.871 mg/mL
Main peak: 99.353 %
C
Concentration: 19.268 mg/mL
Main peak: 99.223 %
D
Concentration: 40.258 mg/mL
Main peak: 99.200 %

## Slide 4
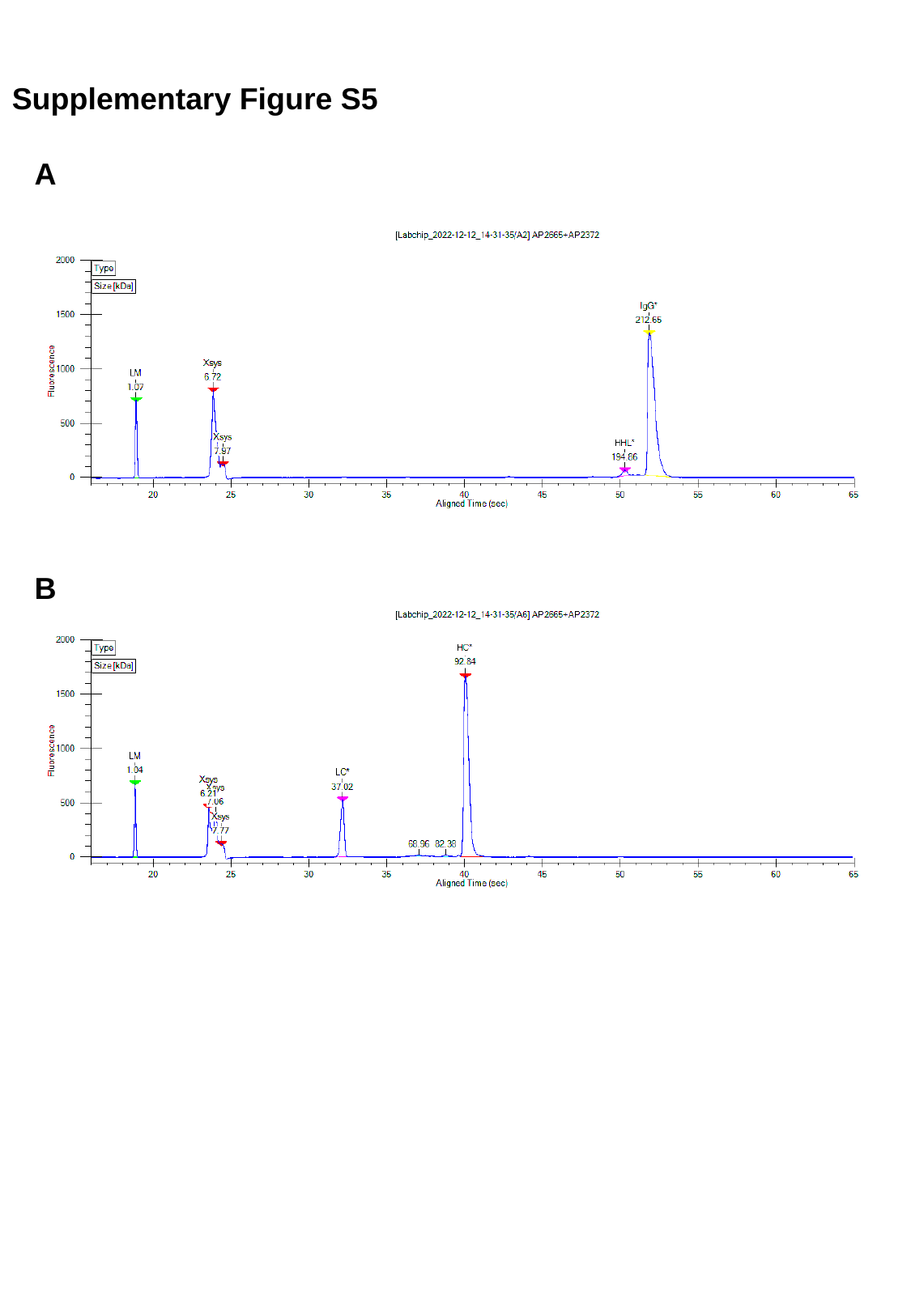

Supplementary Figure S5
A
B

## Slide 5
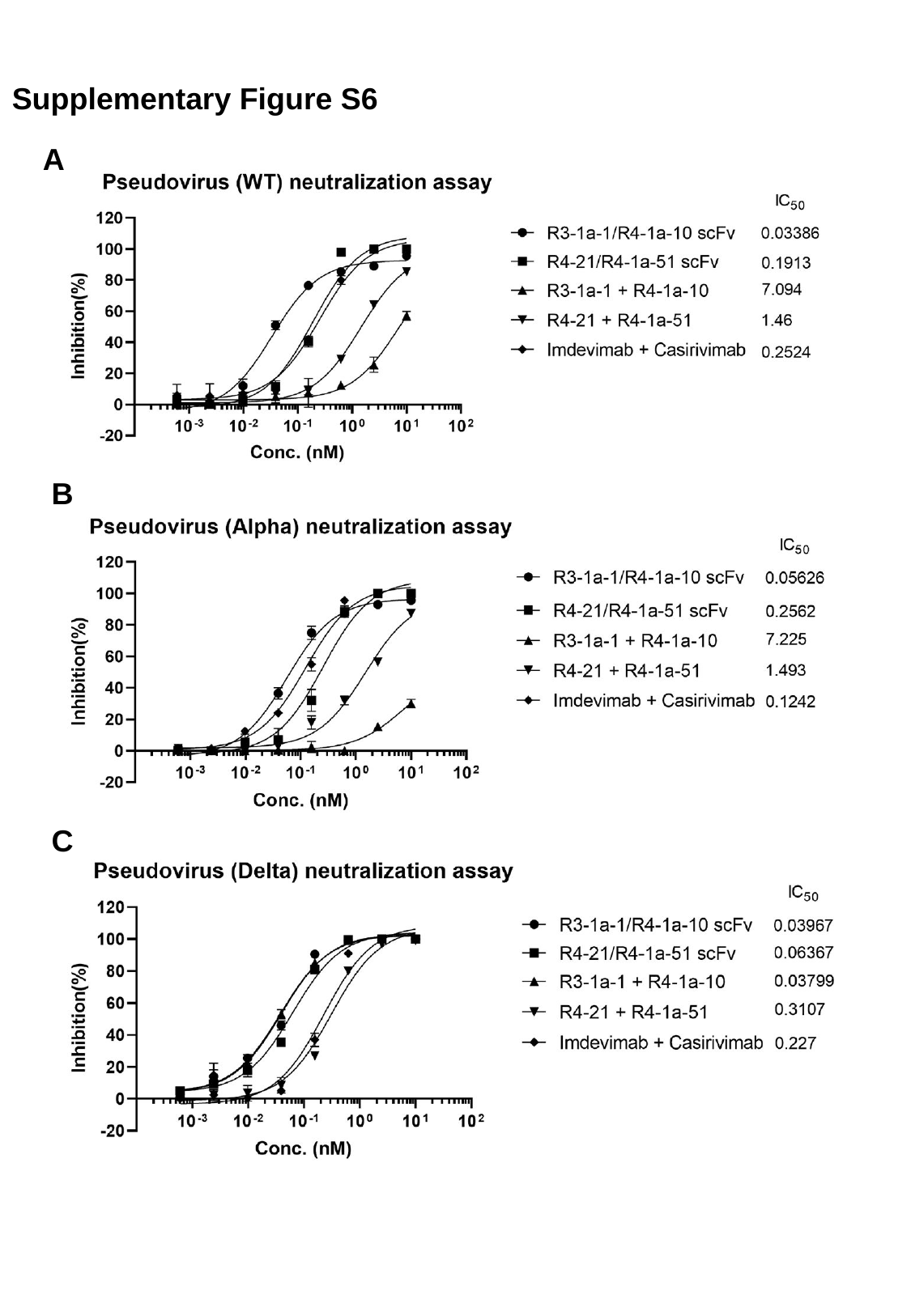

Supplementary Figure S6
A
B
C
