# Supplementary material for: Development of bispecific antibodies with enhanced neutralization activity against tested SARS-CoV-2 Omicron subvariants
Source: Front Immunol. 2026 Jun 17;17:1793368. doi: 10.3389/fimmu.2026.1793368 (PMC13318592; doi:10.3389/fimmu.2026.1793368)
Supplement: Supplementary file 2 [file Table1.docx]

**Supplementary Table S1. RBD amino acid substitutions in recombinant RBD proteins used for antibody binding analyses.**

| **RBD antigen / mutant** | **Amino acid substitutions in RBD** | **Reference/ source** | **Use in this study** |
| --- | --- | --- | --- |
| WT RBD | None relative to ancestral Wuhan-Hu-1 RBD | Manufacturer information | Initial screening |
| E484K | E484K | Manufacturer information | Initial RBD mutant binding screen |
| E484K/N501Y | E484K, N501Y | Manufacturer information | Initial RBD mutant binding screen |
| L452R/E484Q | L452R, E484Q | Manufacturer information | Initial RBD mutant binding screen |
| L452R/T478K | L452R, T478K | Manufacturer information | Initial RBD mutant binding screen |
| Omicron B.1.1.529 / BA.1 RBD | G339D, S371L, S373P, S375F, K417N, N440K, G446S, S477N, T478K, E484A, Q493R, G496S, Q498R, N501Y, Y505H | (Liu et al., 2022) | Binding and kinetic analyses |
| Omicron BA.2 RBD | G339D, S371F, S373P, S375F, T376A, D405N, R408S, K417N, N440K, S477N, T478K, E484A, Q493R, Q498R, N501Y, Y505H | (Cao et al., 2022) | Binding and kinetic analyses |
| Omicron BA.4 / BA.5 RBD | G339D, S371F, S373P, S375F, T376A, D405N, R408S, K417N, N440K, L452R, S477N, T478K, E484A, F486V, Q498R, N501Y, Y505H | (Cao et al., 2022) | Binding and kinetic analyses |

**References**

Cao, Y., Yisimayi, A., Jian, F., Song, W., Xiao, T., Wang, L., Du, S., Wang, J., Li, Q., Chen, X., Yu, Y., Wang, P., Zhang, Z., Liu, P., An, R., Hao, X., Wang, Y., Wang, J., Feng, R., Sun, H., Zhao, L., Zhang, W., Zhao, D., Zheng, J., Yu, L., Li, C., Zhang, N., Wang, R., Niu, X., Yang, S., Song, X., Chai, Y., Hu, Y., Shi, Y., Zheng, L., Li, Z., Gu, Q., Shao, F., Huang, W., Jin, R., Shen, Z., Wang, Y., Wang, X., Xiao, J., and Xie, X.S. (2022). BA.2.12.1, BA.4 and BA.5 escape antibodies elicited by Omicron infection. *Nature* 608**,** 593-602.

Liu, L., Iketani, S., Guo, Y., Chan, J.F., Wang, M., Liu, L., Luo, Y., Chu, H., Huang, Y., Nair, M.S., Yu, J., Chik, K.K., Yuen, T.T., Yoon, C., To, K.K., Chen, H., Yin, M.T., Sobieszczyk, M.E., Huang, Y., Wang, H.H., Sheng, Z., Yuen, K.Y., and Ho, D.D. (2022). Striking antibody evasion manifested by the Omicron variant of SARS-CoV-2. *Nature* 602**,** 676-681.
